# Apixaban Monitoring: The Jury Is Still Out∗

**DOI:** 10.1016/j.jacadv.2022.100038

**Published:** 2022-05-14

**Authors:** Michael W. Rich

**Affiliations:** Division of Cardiology, Department of Medicine, Washington University School of Medicine, St. Louis, Missouri, USA

**Keywords:** anticoagulants, apixaban, atrial fibrillation, DOAC monitoring, geriatric cardiology

**Figure:**
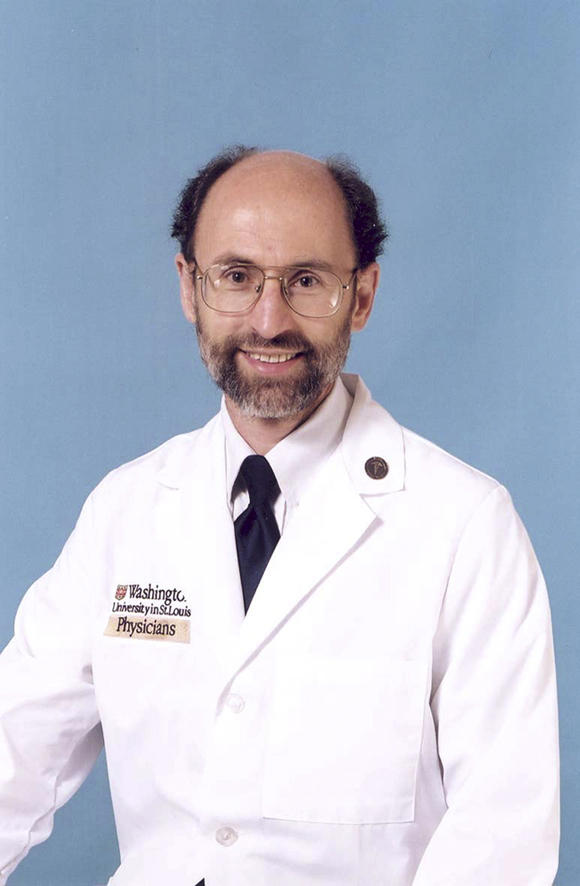

Current guidelines recommend using a direct-acting oral anticoagulant (DOAC) in preference to warfarin in patients with nonvalvular atrial fibrillation (NVAF) and a CHA_2_DS_2_-VASc score of 2 or higher.^1^ This recommendation is based on consistent findings from a series of large randomized trials demonstrating equivalent or superior efficacy of DOACs relative to warfarin in preventing stroke coupled with a lower risk for major bleeding, including intracranial hemorrhage. Clinically, DOACs are more convenient than warfarin for both clinicians and patients because they do not require routine monitoring and they have far fewer interactions with other medications and foods.

Yet, despite the favorable risk-benefit profile of DOACs, patients taking these medications remain at risk for stroke and bleeding complications. In the ARISTOTLE (Apixaban for Reduction In Stroke and Other ThromboemboLic Events in Atrial Fibrillation) trial, for example, patients randomized to apixaban experienced an annual stroke rate of 1.19% and an annual rate of major bleeding of 2.13%.^2^ In patients taking warfarin, it is well established that a subtherapeutic international normalized ratio (INR) is associated with an increased risk for stroke, while the risk for major bleeding rises progressively as the INR exceeds the therapeutic range.^3^ This suggests that some stroke events in patients taking DOACs may be linked to inadequate anticoagulation and that some bleeding events may be associated with excess anticoagulation. This in turn invites the question of whether monitoring DOAC levels, at least in selected patients at high risk for stroke or bleeding, would translate to improved outcomes for patients with NVAF.

In this issue of *JACC: Advances*, Thomas et al^4^ sought to inform this question by measuring peak and trough serum levels of apixaban in 115 older adults (mean age: 80 years, 40% women) with NVAF and increased risk for stroke, as evidenced by a median CHA_2_DS_2_-VASc score of 5. The authors used the reference range reported in the ARISTOTLE trial to classify apixaban levels as being below the fifth percentile (potentially subtherapeutic), above the 95th percentile (potentially supratherapeutic), or within the range.^2^ They then examined the proportion of patients in each of these categories as a function of apixaban dose (5 mg or 2.5 mg twice daily), adherence to labeling recommendations for dosing, and sex. The main findings were that among 83 patients who received the 5-mg dose in accordance with labeling, peak apixaban concentrations were within the range in 72 patients (86.7%), but 6 of 30 women (20%) were above the reference range and 5 of 58 men (8.6%) were below it. Among 11 patients taking the 2.5-mg dose as recommended by labeling, 1 woman was above the range and 4 women were below it. Among 13 patients taking apixaban 2.5 mg without meeting standard criteria for the lower dose, 1 man was above the range, while 1 woman and 4 men were below the range (ie, 38.5% of patients were below the range). Notably, of 3 patients taking 5 mg despite qualifying for the lower dose, all 3 were within the range for both peak and trough levels. In summary, dosing apixaban at 5 mg twice daily in accordance with labeling led to excess levels in 20% of older women, and the use of the 2.5-mg dose led to levels below the range in 37.5% of patients, regardless of whether dosing was in alignment or in conflict with labeling recommendations.

This study represents an important contribution to the literature. Atrial fibrillation is the most common chronic heart rhythm disorder, and more than half of patients in the United States with atrial fibrillation are ≥75 years of age.^5^ Older patients are at an increased risk for both stroke and bleeding, so optimal dosing of anticoagulants is crucial for maximizing benefit and minimizing harm. Apixaban is the most widely prescribed DOAC for NVAF in both the US and United Kingdom, including older adults.^6^ And although 31% of patients in the ARISTOTLE trial were ≥75 years of age, older patients enrolled in clinical trials tend to be “healthier,” with less comorbidity and better function than older individuals encountered in routine clinical practice.^7^ In the Medicare population, more than 55% of patients with atrial fibrillation have 5 or more comorbid conditions^8^; similarly, geriatric syndromes, including cognitive impairment and frailty, are also highly prevalent. Thus, findings from even well-designed clinical trials may not apply to “real-world” older adults due to an age-associated alteration in the risk-benefit balance. The findings of Thomas et al provide evidence to support this concern, as 20% of women treated with apixaban 5 mg twice daily—as directed by guidelines and recommended by labeling—had levels above the reference range, potentially exposing them to an increased risk for bleeding. Conversely, 37.5% of patients treated with apixaban 2.5 mg twice daily had levels below the reference range, potentially placing them at an increased risk for stroke or systemic embolization. While this study is too small to offer meaningful insights into clinical outcomes, it provides a strong rationale for future studies designed to examine the association between apixaban levels (and DOAC levels more broadly) and key clinical outcomes, especially in high-risk populations such as older adults.

Another important observation from this study was that female sex, creatinine clearance, and the number of prescribed medications were independent predictors of apixaban levels. Among patients taking the 5-mg dose as recommended by labeling, peak apixaban levels were 42.5% higher in women than in men, while trough concentrations were 28% higher. Notably, age, weight, race, and frailty were not associated with apixaban levels by multivariable analysis. Based on the ARISTOTLE trial,^2^ reduced dose apixaban (ie, 2.5 mg twice daily) is recommended for patients who fulfill at least 2 of 3 criteria—age ≥80 years, weight ≤60 kg, and serum creatinine ≥1.5 mg/dL. Yet, in the current study, neither age nor weight was associated with apixaban levels. Moreover, although apixaban levels varied as a function of creatinine clearance, this represents a more accurate metric than serum creatinine, which, as noted by the authors, is not a reliable indicator of renal function in older adults. Thus, the study raises concern about the utility of current criteria for determining which patients are most suited to receive a reduced dose of apixaban.

## Study Limitations

The principal limitations of the study are the small sample size and lack of clinical outcomes. Although women were well represented, there were only 11 African Americans, 6 Asians, and no Hispanic patients. The generalizability of the findings to a broader and more diverse population, including younger patients, is unknown.

## Clinical implications

Without evidence demonstrating improved outcomes, such as fewer stroke events and/or less bleeding, in patients with DOAC dosing based on serum levels, the direct clinical implications of this study are limited. However, there are data to suggest that higher DOAC levels are associated with an increased risk for major bleeding^9^^,^^10^ and that underdosing is associated with an increased risk for thromboembolic events.^11^ Taken together, these studies imply that monitoring DOAC levels could lead to improved outcomes, although additional research is clearly needed. The current study also provides strong support for avoiding the 2.5-mg dose in patients who do not meet criteria for the lower dose, especially men. While this message is not new, it bears emphasis since underdosing is relatively common in current practice, occurring in approximately 9% of patients treated with DOACs.^11^^,^^12^

## Future research

Additional studies are needed to better define the role of monitoring DOAC levels as a means for optimizing dosing and reducing both embolic events and major bleeding in patients with NVAF. Such studies might preferentially target older patients due to their higher risk for adverse outcomes but should enroll a racially and ethnically diverse population representative of older patients in clinical practice, including those with multimorbidity, polypharmacy, and geriatric syndromes, as well as community-dwelling individuals and nursing home residents. Once these studies have been completed, the jury will be better positioned to pass judgment on the utility of DOAC monitoring.

## Funding support and author disclosures

The author has reported that he has no relationships relevant to the contents of this paper to disclose.
